# Potential lipid-based strategies of amphotericin B designed for oral administration in clinical application

**DOI:** 10.1080/10717544.2022.2161671

**Published:** 2023-01-05

**Authors:** Xiaoming Zhong, Jianqiong Yang, Hongyan Liu, Zhiwen Yang, Ping Luo

**Affiliations:** a Department of Oncology Radiotherapy, Jiangxi Cancer Hospital, Nanchang, China; b Department of Clinical Medicine Research Center, The First Affiliated Hospital of Gannan Medical University, Ganzhou, China; ;; c Department of Pharmacy, Shanghai Songjiang District Central Hospital, Shanghai, China; d Department of Breast surgery, Nanchang Third Hospital, Nanchang, China

**Keywords:** AmB, oral formulation, clinical application, cochleates, encochleates, emulsions

## Abstract

Amphotericin B (AmB) is regarded as a first-line therapy against life-threatening invasive fungal infections. Due to its poor oral bioavailability, AmB is restricted to intravenous administration in clinical practice. As science continues to move forward, two lipid-based formulations are successfully developed for oral AmB administration, currently undergoing phase I clinical trials. Encouragingly, lipid-AmB conjugates with emulsions also exhibit a better bioavailability, which may be another strategy to design oral AmB formulation in clinical practice. Thus, this review mainly focused on the two lipid-based formulations in clinical trials, and discussed the potential perspectives of AmB-lipid conjugation-loaded nanocochleates and emulsions.

## Introduction

AmB is regarded as a first-line drug in treating the serious systemic fungal infections. Due to the poor water solubility and low membrane permeability, AmB with the parenteral formulations is most frequently used in clinical practice (Groll et al., 2019). Intravenous injection formulations, including Fungizone®, Abelcet®, Ambisome®, Amphocil®, have been marketed for many decades. However, AmB toxicity and costs associated with the lipid formulations hampered its widespread usage in hospitals (Suberviola, 2021).

Oral administration is the most widely used route and most readily accepted form for the patients (Gaba et al., 2015). Unfortunately, none of the commercial AmB formulations is produced by now. It is difficult in reaching efficacious bioavailability when orally administered. In recent years, various nanotechnology-based drug delivery systems, such as solid lipid nanoparticles (Chaudhari et al., 2016), lipid conjugates (Faustino and Pinheiro, 2020), micelles (Risovic et al., 2003), emulsions (Wasan et al., 2009), self-emulsifying drug delivery systems (Gershkovich et al., 2010), cubosomes (Yang et al., 2014), polymer lipid hybrid nanoparticles (Jain et al., 2012), PLGA nanoparticles (Italia et al., 2009), polymeric nanoparticles (Verma et al., 2011), carbon nanotubes (Benincasa et al., 2011), and lipid–polymer hybrid nanoparticles (Faustino and Pinheiro, 2020), have been investigated for enhancing AmB bioavailability. They all have in common to overcome the shortcoming of parenteral administration, greatly to decrease its toxicity, and improve its efficacy. Of note, almost all of oral AmB formulation only stay in the stage of animal experiment research, lack of further clinical study (Chaudhari et al., 2016; Faustino and Pinheiro, 2020; Risovic et al., 2003; Wasan et al., 2009; Gershkovich et al., 2010; Yang et al., 2014; Jain et al., 2012; Italia et al., 2009; Verma et al., 2011; Benincasa et al., 2011).

Due to a series of clinical trial failure, it seems to be impossible to develop a successful oral formulations of AmB. Along with the in-depth understanding in drug-delivery technologies, scientists with the positive attitude reassess and develops AmB formulation for oral administration (Thornton and Wasan, 2009). AmB-loaded cochleates and emulsions have obtained the encouraging results over the last decades, exhibiting the possibility via the oral route in clinical practice (Thornton and Wasan, 2009). Thereby, our review mainly focused on the AmB lipid formulation, undergoing oral clinical trials with a better bright prospect in an industrial application. First, molecular state of AmB in lipid environments was important for understanding its biological activity. Second, AmB-loaded cochleates and emulsions were currently undergoing clinical trials with a promising alternative to parenteral therapy. Finally, a prodrug of AmB-lipid conjugation loaded in lipid formulation may be another strategy for developing oral product in clinical practice

## The importance of oral AmB product

Due to poor absorption across the gastrointestinal tract, many current pharmaceutical therapies are parenteral in clinical practice. Intravenous drug therapies require attendance at hospital, resulting in an inconvenience for patients. Intravenous formulation necessitate the presence of trained medical personnel, and increase a financial burden due to the use of infusion equipment (Khatoon et al., 2017; Mir et al., 2017).

Intravenous AmB has been limited by numerous significant toxicities, including dose-dependent nephrotoxicity, anemia, and infusion-related reactions (Skipper et al., 2020; Cuddihy et al., 2019; Hnik et al., 2020). Furthermore, intravenous AmB require sophisticated electrolyte monitoring tools for these toxicities, which may be an insurmountable barrier and preclude use of this medication in developing countries, especially in the African country with large populations at risk (Farmakiotis et al., 2013; Messori et al., 2013).

To overcome these challenges, it is necessary for the development of an oral formulation of AmB that is easy to administer, cost effective, and nontoxic yet retains excellent pharmacological activity in the clinical application (Din et al., 2017; Zeb et al., 2020; Radwan et al. 2017; Kim et al., 2018).

## Molecular state of AmB in lipid environments

### AmB molecular characterizations

**Figure 1.**
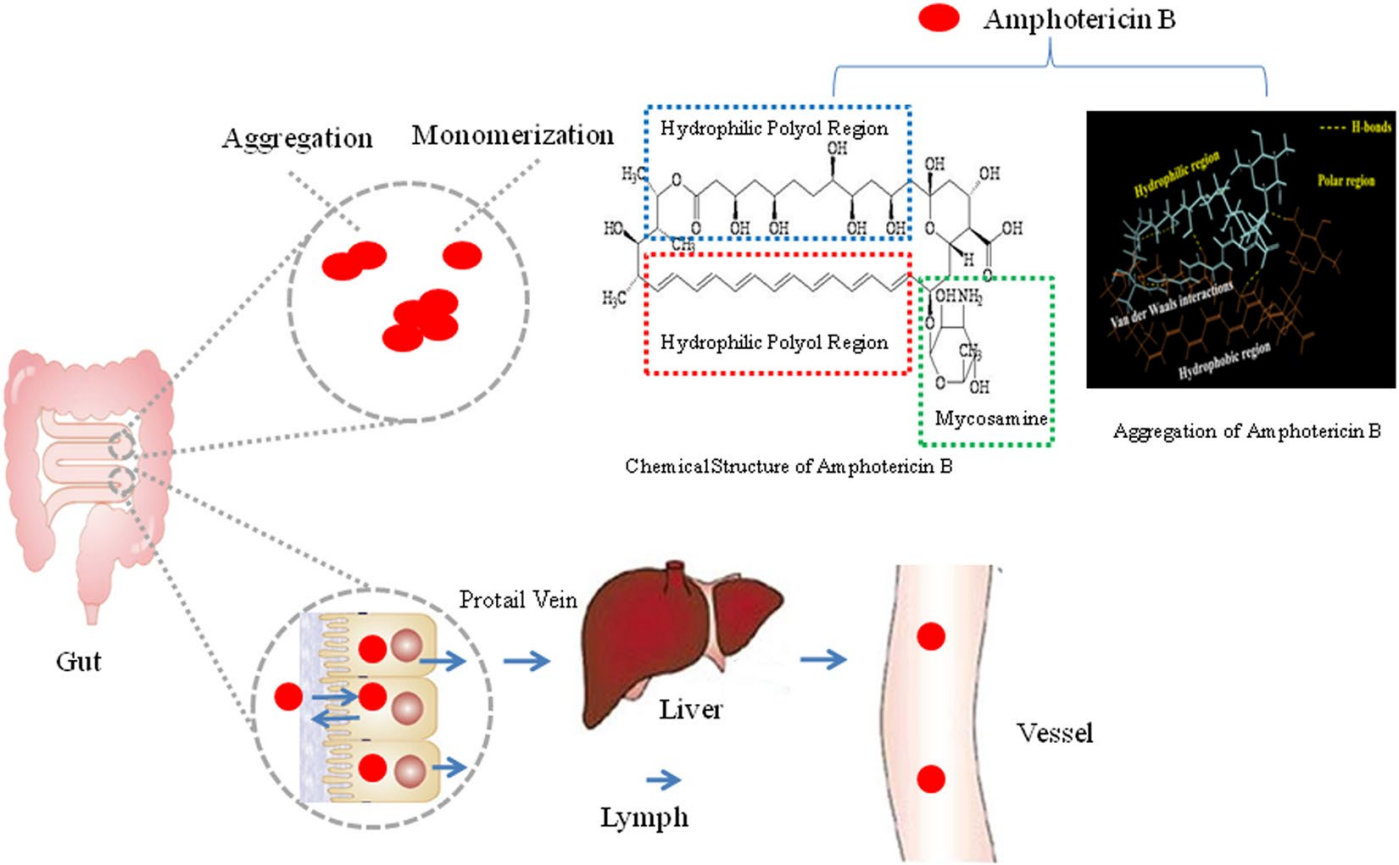
The absorption process of AmB molecule in gastrointestinal tract.

AmB has a molecular weight of 924 Da (Cuddihy et al., 2019; Liu et al., 2017). Molecular structure of AmB is comprised of a macrolactone ring and non-polar heptene group (Figure 1). The ring is β-glycosylated at C19 with a mycosamine group, exhibiting an almost flat chromophore with seven conjugated double bonds in the trans conformation. At C13 and C17, the macrolactone ring also contains a hemiketal ring. The presence of an amino group in the mycosamine head group and a carboxyl group at C16 determines the amphoteric nature of AmB (Cuddihy et al., 2019; Liu et al., 2017). Additionally, the specific three-dimensional structure of this molecule is determined to own hydrophobic and hydrophilic regions, further conferring its amphipathic properties (Cuddihy et al., 2019; Liu et al., 2017). Consequently, AmB is responsible for poorly soluble in highly polar and nonpolar solvents (Cuddihy et al., 2019; Liu et al., 2017; Ciesielski et al., 2016).

AmB molecules are divided into three different aggregation states in aqueous media, including monomeric monomers, oligomers or oligo-aggregates, and poly-aggregates (Torrado et al., 2013; Zielińska et al., 2016). Due to its amphiphilic structure and low solubility, AmB tends to aggregate in aqueous solution. AmB monomers exist in water at a very low concentration of 5 × 1 0 ^−7^–1 0 ^−4 ^M (Torrado et al., 2013; Zielińska et al., 2016). Above this concentration, AmB molecules occur mostly in the self-aggregated form. Generally, the monomers self-assembled into oligomers or oligo-aggregates form and these into poly-aggregates (Torrado et al., 2013). Oligo-aggregates are usually defined as dimers, even 4 and 8 molecules aggregation of AmB (Zielińska et al., 2016). AmB exhibits four characteristic peaks in the UV absorption spectra i.e., around 407 nm, 385 nm, 365 nm and 344 nm (Torrado et al., 2013; Zielińska et al., 2016). The ratio of the intensity of peak I to peak IV determines the aggregation state of AmB molecules. As reported, the lower ratio of the intensity represents the monomeric state, while its higher ratio confirms molecular aggregates (Thanki et al., 2018).

Molecular behavior of AmB poses a great challenge to develop an oral formulation. Due to the presence of zwitterionic nature, structural asymmetry, and amphiphilicity, AmB shows poor aqueous solubility (<1 mg/L) at the physiological pH, which can cause a significant precipitation in the gastrointestinal tract (Shim et al., 2011). Also, the drug with the polyene chain shows highly unstable at acidic or alkaline pH, leading to an easily degradation under the acidic environment in the stomach upon oral administration (Liu et al., 2017). Additionally, AmB has a poor permeability in the intestinal tract due to the high molecular weight, low solubility, and preferential interaction with the cellular membrane. The oral bioavailability of AmB exhibits a very poor data, ranging from 0.2 to 0.9% (Liu et al., 2017; Volmer et al., 2010).

## Aggregated state of AmB in lipidic excipients

AmB can interact with ergosterol located on fungal lipid membranes to disrupt and create pores, showing its antifungal activity (Maertens et al., 2022). At the same time, AmB–cholesterol interactions with cell components from mammalian cells also take place, leading to toxicity. Substantial researches have proposed that the toxicity of AmB is directly related to its aggregation state (Martin-Loeches et al., 2020; Adler-Moore et al., 2019; Starzyk et al., 2014). The efficacy/toxicity ratio can be modulated by controlling the monomeric and aggregation state (Kamiński, 2014). To better explore the effects of AmB aggregation in biological systems, it is important to understand how AmB interacts with lipids.

Some experiments have been conducted to investigate the aggregation of AmB in lipid excipients. The strategy to use lipids for stabilizing the monomer state of AmB had focused on not only limiting the hydrophobic interactions of the drug, but also masking the polar regions of the macrolide ring, and increasing the solubilization capacity (Das and Devarajan, 2020). Fujii et al. found that AmB occurred in self-associated forms when the ratio of AmB to lipid molecules was up to 1:1,000, while AmB below this concentration existed in the lipid bilayer mostly in the monomeric form (Fujii et al., 1997). This study further confirmed the similar finding that aggregated forms of AmB were observed at a concentration of 2.5 mg AmB per 10 mg phospholipid (Hargreaves et al., 2006). Gruszecki et al. reported that AmB forms aggregated as dimers in lipid bilayers containing cholesterol, whereas both monomeric and aggregated forms were present in bilayers containing ergosterol (Diezi and Kwon, 2012). Tween® and Span® series in combination with different oils were investigated for the solubilization capacity of AmB, revealing that they enhanced the drug solubility up to 1000-fold (Silva et al., 2013).

Lipidic excipients are defined as effective approaches to achieve monomer state of AmB, which indicated a promising strategy to reduce the toxicity in mammalian cells and to trigger the selective response against fungal cells (Perrella et al., 2020; Kulkarni et al., 2021). For instance, the toxicity of AmB in Fungizone®, due to the oligomeric form in nanomicelles, was significantly attenuated by mixing with a parenteral fat emulsion (Egito et al., 2002). The absorption spectra analyses showed a stable disperse state of Fungizone/nutritional purpose (intralipid or lipofundin) admixture until the concentration of 5 × 1 0 ^−7 ^M (Egito et al., 2002). Morphological studies revealed that AmB self-association state was changed by binding drug with emulsion droplets (Egito et al., 2002). As a result, the reduced toxicity can be attributed to the presence of AmB monomerization, dependent on the additional concentration of the lipidic emulsion (Egito et al., 2002). Similarly, disaggregation of AmB in Fungizone® was induced by lipidic micelles, revealing a significant decrease in toxicity (Alvarez et al., 2016).

## Lipid systems for the oral delivery of AmB

Lipid vehicles are attractive drug delivery systems to overcome its poor water solubility and improve oral bioavailability of AmB (Perlin, 2004). Moreover, lipid delivery systems upon oral administration are able to avoid infusion-related adverse events, decrease the systemic toxicity, reduce the costs, and improve patient compliance (Karimzadeh et al., 2013; Parvez et al., 2020). Interestingly, after drug is encapsulated into lipid delivery systems, AmB molecule in lipid environments exhibit the characterization of the disaggregation state, depending on the lipid composition (Silva et al., 2013). Lipid-based systems for the potential delivery of oral AmB over the last three years are summarized in Table 1.

**Table 1.** Lipid-based systems for oral AmB delivery in experimental laboratory over the last three years.

Formulation	Composition	Adm. Route	Size (nm)	EE (%)	Dose (mg/kg/day)	Cellular Model	SGF	Antileishmanial Efficacy in mice	Ref.
NLCs	Eu-NLCs	Oral	643.5 ± 41.9	76.9 ± 1.3	N/A	Caco-2	Stability	N/A	(Nimtrakul et al., 2020)
	Cs-SLNs	Oral	373.9 ± 1.41	95.20 ± 3.19	N/A	Caco-2	Stability	N/A	(Parvez et al., 2020)
	VBS-SLNs	Oral	306.66 ± 3.35	97.99 ± 1.6	N/A	J774A.1 macrophage	Stability	N/A	(Singh et al., 2020)
Liposomes	PEGylated liposomes	Oral	125 ± 12	97.9 ± 0.7	5 mg/kg at 2-day intervals for 24 days	N/A	Stability	Efficiency	(Ramos et al., 2022)

Adm. Route: Administration Route; EE: encapsulation efficiency; SGF: Simulated Gastric Fluid; Ref: Reference.

Nanostructured lipid carriers (NLCs) attract more attention due to their ability to promote intestinal absorption and enhance oral bioavailability of AmB (Senna et al., 2018; Ghosh et al., 2019). Pataranapa et al developed a novel NLCs coating with Eudragit® L100-55 (Eu-NLCs) for improving drug stability in gastrointestinal fluids (Nimtrakul et al., 2020). Enteric pH-dependent Eudragit L100-55® as pharmaceutical excipients have been widely applied for enteric-coated tablet as it is soluble in medium of pH > 5.5 (Moustafine et al., 2008). Eu-NLCs possessed a particle size of 550 nm with entrapment efficiency up to 75%, and significantly enhanced AmB water solubility by up to 10-fold compared with the free drug. In fasted state simulated gastric fluid, Eu-NLCs provided a better ability to protect the drug from degradation in gastric fluid (Nimtrakul et al., 2020). However, the work only was performed in vitro, but not in animal or human (Nimtrakul et al., 2020). Shabi et al. prepared the chitosan functionalized NLCs (Cs-SLNs) for enhancing the muco-adhesive property in gastrointestinal tracts (Parvez et al., 2020). Chitosan exhibits greatly muco-adhesive properties that enhance their absorption across gastrointestinal tract, and expedite the transport capacity of nanoparticles (Min et al., 2018). Cs-SLNs had a stable state in various simulated gastro-intestinal fluids, achieved complete internalization in Caco-2 cells, and eventually improved the AmB bioavailability when administered orally (Parvez et al., 2020). Aakriti et al. produced the AmB-loaded NLCs coated with vitamin B12-stearic acid conjugate (VBS-SLNs) (Singh et al., 2020). Stearic acid and vitamin B12 have been used to improve oral bioavailability in the preparation of SLNs (Kumar and Randhawa, 2015; Verma et al., 2016). As a result, VBS-SLNs showed a chemical stability of AmB in simulated gastrointestinal fluids, and complete internalization of the formulation in cellular uptake studies (Singh et al., 2020). Eventually, VBS-SLNs exhibited the increased oral delivery of AmB by enhancing a favorable mucus permeating ability (Singh et al., 2020).

Liposomes, concentric bi-layered structures, are formed by the self-assembly of phospholipids in aqueous media. Guilherme et al. presented the incorporation of AmB into PEGylated liposomes (Ramos et al., 2022). The PEGylated vesicles showed a particle size of 100–130 nm with a high entrapment efficiency up to 95%. Due to the specific interaction of AmB with membrane lipids, AmB in PEGylated liposomes were characteristic of the non-aggregated form. PEGylated liposomes, following a relatively low dose of 5 mg/kg given at 2-day intervals, were effective when given by oral route in a murine model of infection (Ramos et al., 2022). The mechanism is responsible for improving the stability of the drug molecule in gastrointestinal tracts and protecting AmB from acid degradation (Ramos et al., 2022).

## Potential mechanisms for the enhanced gastrointestinal absorption

**Figure 2.**
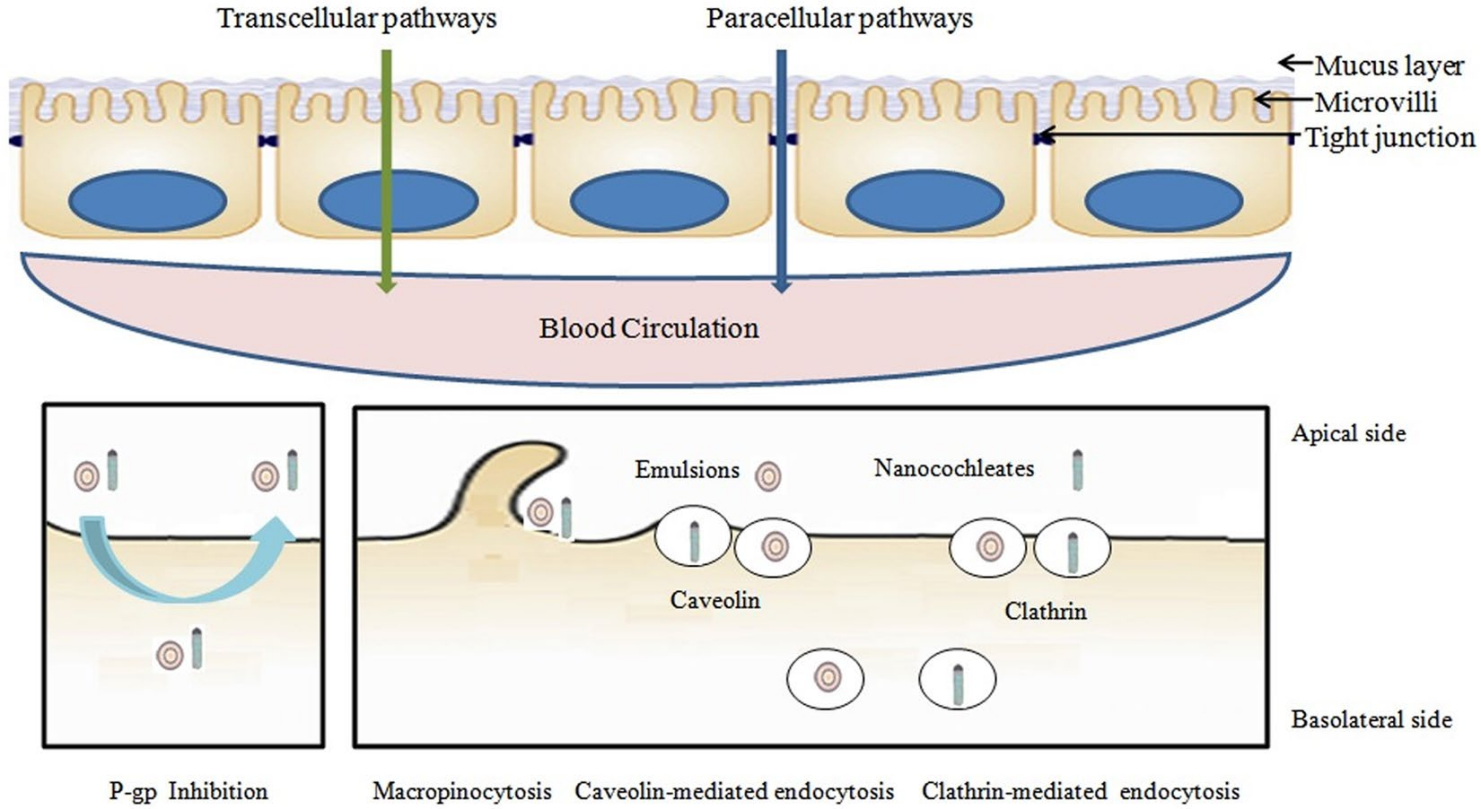
Nanotechnology enhances the oral absorption of poorly water-soluble drugs.

Nanotechnology can improve the oral bioavailability of poorly water-soluble drugs (Figure 2), and address the principal disadvantages of oral administration, including the enhancement of mucoadhesion and cellular uptake, prolongation of residence time, alteration of absorption pathways in the gastrointestinal tract, increase of potential solubilization and superior encapsulation, prevention of metabolic degradation within the gastrointestinal tract, and optimization of chemical materials eligible for nano-medicine (Fonte et al., 2015; Horev et al., 2015; Fatma et al., 2016). To date, how to design, select, and develop oral delivery systems are still a great challenge, which is required for the in-depth understanding of drug physicochemical properties and nanoparticles under physiological conditions. Particle composition, surface properties, size, shape, and function of nanoparticles play an important role in the in vivo fate of nano-drug delivery systems after oral administration (Ma et al., 2017; Ma et al., 2015). Various oral absorption mechanisms, such as enterocytes transport, tight junction modulation, mucoadhesion, receptor mediated endocytosis and transcytosis, phagocytosis, obviously affect drug uptake across the gastrointestinal cellular membrane (Shan et al., 2015).

## Oral lipid formulations of AmB in clinical trials

To our knowledge, several AmB formulations, comprising topical solutions for skin infections, topical pulmonary and intrathecal AmB formulations, and AmB for injection are investigated at different stages of clinical trials (Faustino and Pinheiro, 2020; Zahid et al., 2022). Encouragingly, several AmB formulations for oral administration have entered clinical development (Table 2), including AmB-loaded cochleate, AmB-loaded encochleates, and AmB-loaded emulsions.

**Table 2.** Clinical trials of oral AmB safety and efficacy against antifungal diseases.

Formulation	Phase	Trial identify	Population(n)	Treatment Regimen	Disease	Status	Start/Completion Date
Cochleates	1	N/A	48	A single dose(200, 400, 800 mg)	healthy human	Completed	2008.7–2009.2
Encochleated AmB	3	NCT05541107	270	N/A	Cryptococcal Meningitis	Not recruiting	2023.1–2025.1
	2	NCT02971007	137	200 mg and 400 mg for 5 days	Vulvovaginal Candidiasis	Completed	2016.11–2017.3
	1, 2	NCT04031833	176	N/A	Cryptococcal Meningitis	Recruiting	2019.10–2023.5
	2a	NCT02629419	4	A single dose(200, 400, 800 mg)	Mucocutaneous Candidiasis	Completed	2016.9–2022.8
	2	NCT03187691	N/A	N/A	Antifungal Prophylaxis	Withdrawn	2019.3
	2	NCT03167957	N/A	N/A	Vulvovaginal Candidiasis	Withdrawn	2019.3
Emulsions AmB	1	N/A	32	A single dose(200, 400, 800 mg)	healthy human	Completed	2019.9–2020.4

## AmB-loaded cochleate

Cochleates are the novel nanostructured lipid carriers, formed by the interaction of negatively charged phosphatidylserine and calcium (Bozó et al., 2015). Because of its hydrophobic nature, AmB molecules are localized in the interior lipid layer of cochleates. This unique location could provide effective protection from AmB degradation when it expose to harsh environment in gastrointestinal tract (Santangelo et al., 2000). AmB-loaded cochleates yields potentially prevention of drug exposure, thus reducing drug-related toxicity (Aigner and Lass-Flörl, 2020).

BioDelivery Sciences International (NC, USA) has been developed to a cochleate formulation of AmB (Bioral®) for facilitating oral absorption in preclinical trials (Santangelo et al., 2000; Zarif et al., 2000; Delmas et al., 2002). The oral efficacy of AmB cochleates was investigated in mouse models of both candidiasis and aspergillosis (Santangelo et al., 2000; Zarif et al., 2000; Delmas et al., 2002). Oral AmB cochleates (0.1, 1, 2.5, 5, 10, and 20 mg/kg) were, respectively, applied to treat the disseminated candidiasis infection, showing a complete clearance of fungal load in the lungs at 2.5 mg/kg/day, an obvious reduction of fungus in the kidneys at 2.5 mg/kg/day orally equal to Fungizon® 2 mg/kg intraperitoneal injections, and the 100% survivorship at all doses (Santangelo et al., 2000; Zarif et al., 2000; Delmas et al., 2002). Oral AmB cochleates also indicated a positive antifugal activity in an immunosuppressed mouse model of aspergillosis, decreased fungal tissue burden and reduced the level of mortality after 15 days of oral administration (Santangelo et al., 2000; Zarif et al., 2000; Delmas et al., 2002). In rats and dogs, AmB 15, 30 and 45 mg/kg oral route once-daily ×7 days was well tolerated, without mortalities or clinical abnormalities. Plasma Cmax was 73 and 104 ng/ml and AUC was 633 and 1181nghr/ml for rats and dogs, respectively. No significant toxicological differences or histopathological findings were observed. No adverse events were observed at up to 45 mg/kg in males and females of both species (Santangelo et al., 2000; Zarif et al., 2000; Delmas et al., 2002).

In February 2009, oral AmB-loaded cochleates (Bioral®) developed by BioDelivery Sciences International released an interesting clinical outcome in phase I trial, indicating a positive antifungal therapy equivalent to intravenous therapy, and well-tolerated clinical data without meaningful changes in safety values including those associated with renal functions (Thornton and Wasan, 2009). Pharmacokinetic evaluation showed plasma concentrations were comparable to those seen in prior animal studies using the same formulation (Thornton and Wasan, 2009). This was the first time that AmB cochleates stated a big step forward for an oral administration.

However, Biodelivery Sciences International seems to stop the study of oral AmB-loaded cochleates, and the further clinical trial has not been explored in the past ten years (Lipa-Castro et al., 2021). The exact reason for this remains unclear due to the absence of the relative information from Biodelivery Sciences International. Antonio et al believed that cochleate nanocarriers composed of the synthetic 18-carbon monounsaturated 1,2-dioleoyl-sn-glycero-3-phospho-L-serine was expensive, limiting the clinical use. They developed cochleate nanocarriers using a naturally occurring phospholipid, instead of costly synthetic lipid (Lipa-Castro et al., 2021). As expected, there are still major challenges that AmB cochleates were produced for oral formulation.

## AmB-loaded encochleates

**Figure 3.**
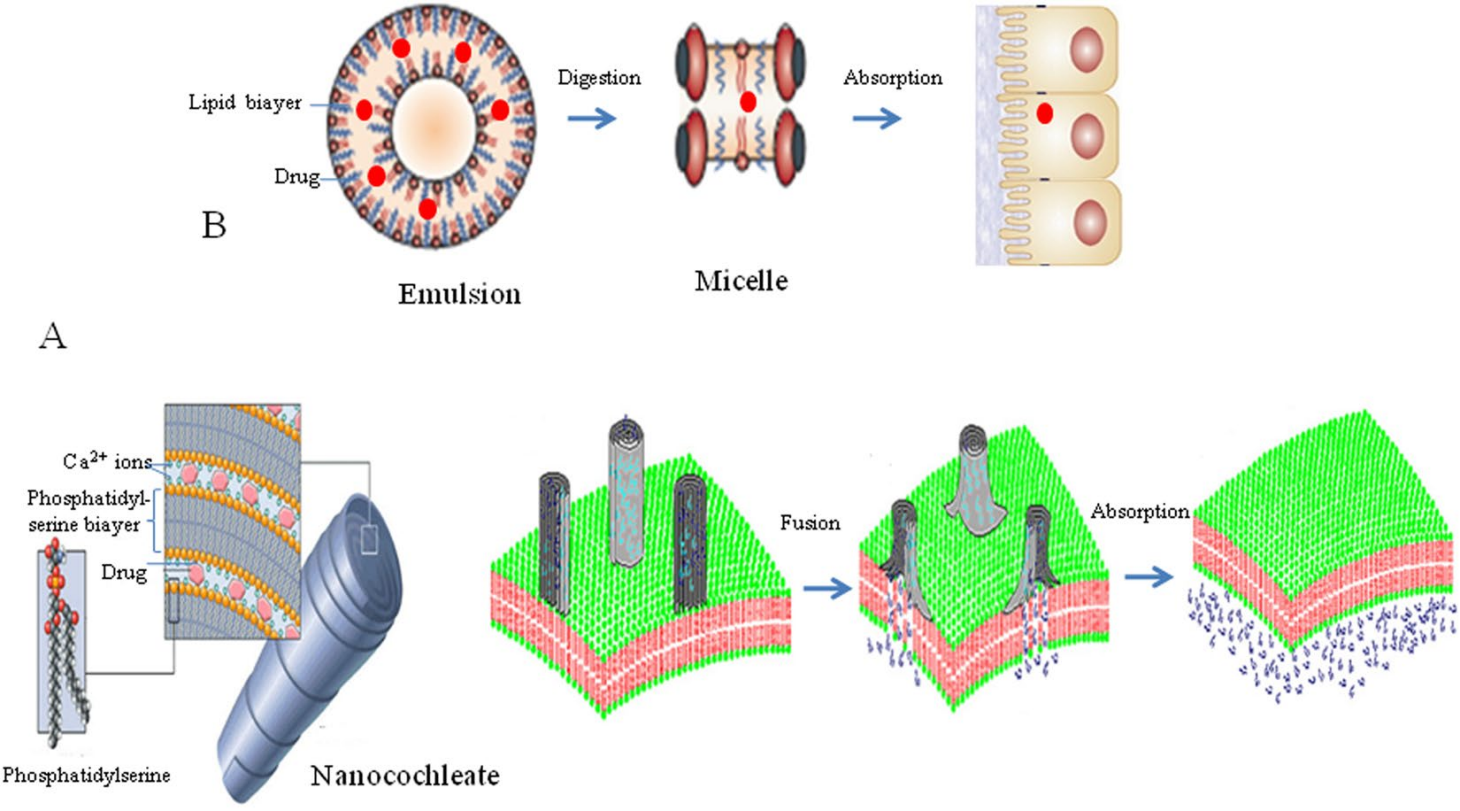
The absorption process of oral AmB formulation in gastrointestinal tract. A: AmB-loaded encochleate; B: AmB-loaded emulsion.

Compared with the AmB cochleates produced by Biodelivery Sciences International, AmB encochleates had a slight difference in preparation methods. AmB deoxycholate, instead of AmB methanol or NaOH solution, was encapsulated in cochleates, which was defined as AmB encochleates (Skipper et al., 2020; Lu et al., 2019). Collaborating with the National Institutes of Health/National Institute of Allergy and Infectious Disease (NIH/NIAID), Matinas BioPharma (NC, USA) is on the way to develop an encochleated AmB formulation (Figure 3) (Chaudhari et al., 2016; Matinas Biopharma, 2021), currently undergoing phase I and II clinical trials (Skipper et al., 2020; Kibathi et al., 2018; U.S. National Library of Medicine., 2015). The United States Food and Drug Administration (FDA) has granted Qualified Infectious Disease Product (QIDP) as well as Fast Track status designation to AmB encochleates with the potential for Orphan Drug Designation (Aigner and Lass-Flörl, 2020). Moreover, several AmB formulations, comprising cochleates, encochleates and emulsions, are investigated in oral clinical trials. To our knowledge, AmB encochleates are the only available oral formulation, which has entered phase II clinical trials and planned to do phase III clinical trials (Aigner and Lass-Flörl, 2020).

Some clinical trials have provided data about the efficacy, safety of encochleated AmB. First, one hundred and thirty seventh patients with severe vulvovaginal candidiasis were enrolled for assessing the safety, efficacy, tolerability, and pharmacokinetic study of encochleated AmB (Clinical Trials.gov registration no. NCT02971007). All these participants upon encochleated AmB doses ranging from 200 to 400 mg had a positive clinical response of 50%−85% clinical improvement. Encochleated AmB at 200 to 400 mg/day could reach AmB plasma concentration of 160 ng/mL within 24 hrs. During the 1-year study period, no serious adverse events were observed in liver, kidney, or hematologic disorders, exhibiting the excellent safety at 400 mg/day administration (Skipper et al., 2020; Kibathi et al., 2018; U.S. National Library of Medicine, 2020). Second, four patients with chronic mucocutaneous candidiasis were enrolled in clinical tials (Clinical Trials.gov registration no. NCT02629419). All these participants upon oral encochleated AmB had a positive clinical response, with no evidence of drug side effects apart from slight gastrointestinal discomfort (Skipper et al., 2020; U.S. National Library of Medicine, 2020). Third, one hundred and sixty seven patients with HIV-positive Ugandans were enrolled for the safety, tolerability, and dose of oral encochleated AmB (ClinicalTrials.gov under registration no. NCT04031833). The trial was divided into two sequential section, with twenty-seven patients in phase IA and nine patients in phase IB. Phase IA was designed to assess a single ascending dose regimen of encochleated AmB for 3 days, and phase IB evaluate the tolerability of multiple-dose cohort for 7 days. Phase IA cohort showed the similar pharmacokinetic data across the different doses, area under curve of 2,200 ng•h/ml, half-life time of 48 h, and maximum plasma concentration of 59.2 ng/ml, while phase IA cohort indicated the plasma concentration of 74.8 ng/ml at 24 h and 91.1 ng/ml at the day 7. These two trials established that encochleated AmB at doses of up to 1.5 g/day for 7 days was generally safe and tolerated in all these participants (Skipper et al., 2020; U.S. National Library of Medicine, 2020). Fourth, based on the above successful phase I and II data, Matinas BioPharma plan to move oral encochleated AmB to a phase III antifungal efficacy trial for cryptococcal-infected meningitis patients in Jan 2023 (U.S. National Library of Medicine, 2022).

However, it is still uncertain whether oral encochleated AmB can be successfully developed in the market. A planned phase II trials in 2020, studying the pharmacokinetics and safety of oral encochleated AmB for prevention of fungal infections in approximately 30 patients undergoing chemotherapy for lymphoblastic leukemia and acute myelogenous, has been withdrawn (U.S. National Library of Medicine, 2020). Likewise, another study of oral encochleated AmB (Clinical Trials.gov registration no. NCT03167957) is withdrawn before participants are enrolled (U.S. National Library of Medicine, 2020).

## AmB-loaded emulsions

iCo Therapeutics Inc. (BC, Canada) successfully developed a lipid-based self-emulsifying drug delivery system (SEDDS) for oral AmB administration (Figure 3) (Wasan et al., 2009; Cuddihy et al., 2019; Hnik et al., 2020; Wasan, 2020; Ibrahim et al., 2013). Peceol® excipient, composed of mono- and di-glycerides, was approved by the FDA, which could enhance a 50-fold solubility of AmB (Adhikari et al., 2017). Subsequently, AmB was incorporated into the Peceol® lipid-based formulation, namely, iCo-019 formulation. On June 27th, 2018, iCo Therapeutics completed phase 1 trials of iCo-019, announced a positive clinical outcome with a better safety, tolerability, and bioavailability (Hnik et al., 2020; Wasan, 2020; iCo Therapeutics Inc., 2018).

The Phase 1 study in Australian was a randomized, double-masked, placebo-controlled, single dose ascending study in thirty-two healthy subjects who are healthy male and non-pregnant female subjects between 18 and 55 years of age. All healthy subjects were divided into four group. Each group contained eight subjects where two subjects received the placebo and six subjects received the investigational product (Hnik et al., 2020; iCo Therapeutics Inc., 2018). All these participants, including the highest dose of 800 mg, were well tolerated without serious adverse events (gastrointestinal tract, liver, kidney toxicity) and drug-related adverse events (Hnik et al., 2020; iCo Therapeutics Inc., 2018). Additionally, iCo-019 at the different doses achieved favorable AmB pharmacokinetics. At the 400-mg dose of iCo-019, maximum plasma concentration of 28.4 ng/ml, peak time of 6.0 h, half-life time of 39 h, and area under curve of 2029 ng•h/ml were observed. In an ascending single dose of treatment, iCo-019 at the 400-mg dose exhibited an approximate doubling of the AUC measure. The absolute bioavailability of AmB from oral iCo-019 reached about 2%–3% (Hnik et al., 2020; iCo Therapeutics Inc., 2018). Of note, fundamentally unlike parenteral AmB, iCo-019 exhibited a prolonged half-life and sustained drug exposure, leading to an increased tissue accumulation against leishmaniasis and systemic fungal infections. Interestingly, pharmacokinetics of iCo-019 in human clinical trials was superior to that of the encochleated AmB (Hnik et al., 2020; iCo Therapeutics Inc., 2018).

Given a successful phase I human clinical trials, iCo Therapeutics are planning to initiate a phase II in early 2020 (iCo Therapeutics Inc., 2018; Adhikari et al., 2017). However, due to the current COVID-19 events, iCo Therapeutics in 2020 announce that it will delay the start of the anticipated Phase 2 trial of iCo-019 (iCo Therapeutics Inc, 2020).

## Lipid conjugation as a promising strategy

Lipid–drug conjugation were designed to propose a delivery system that can increase the oral bioavailability of drug. The conjugation of lipid to drug resulted in a high hydrophobicity of drug for improving its permeability and stability in the gastrointestinal tract. The lipids including phospholipids, long-chain fatty acids such as oleic acid (OA), stearic acid, and lipoamino acids covalently linked to drugs, forming lipid-drug conjugation (Adhikari et al., 2017). The potential of lipid-drug conjugation was widely used for a variety of drugs (Arouri et al., 2013).

OA, a known intestinal permeation enhancer, was selected to be the optimal lipid for covalent conjugation with AmB (Khatoon et al., 2017). First, the aggregated state of AmB-OA was studied, indicative of differential aggregation behavior as that of AmB (Khatoon et al., 2017). AmB-OA at concentration of 10 µM was a similar trend of increasing aggregation as compared to AmB. Notably, in contrast to a head-to-tail arrangement in AmB aggregation, AmB-OA dimers were accommodated in a head-to-head arrangement. AmB-OA aggregation demonstrated the significantly higher hydrophobicity compared with that of AmB. AmB-OA in the aggregated state was found to be selectivity for ergosterol over cholesterol, but not AmB aggregation. The concentration-dependent aggregation of AmB-OA did not induce nephrotoxicity in vivo or hemolytic toxicity in vitro (Khatoon et al., 2017). Second, the potential of AmB-OA for oral therapy was systematically assessed in vitro, indicating an increased uptake of AmB (Thanki et al., 2019). AmB-OA showed less than 5% degradation in 2 h exposed to simulated gastrointestinal fluids, while degradation of AmB was up to 64%. The results revealed that AmB-OA substantially improved AmB stability in a gastric environment. AmB-OA displayed an approximately 3.11-fold increase across the Caco-2 cell monolayer as compared to AmB, due to cumulatively favoring these molecular property such as the log P, and hydrophobicity. Metabolism of AmB-OA into AmB was higher than 80% in the presence of liver homogenates, which warranted AmB-lipid bioconversion upon oral administration (Thanki et al., 2019). AmB-OA in vivo AmB-OA to rats after oral administration at a dosage of 10 mg/kg showed an ∼4.88-fold increase in AUC_Tot_, a 3.13-fold increase in the Cmax compared to AmB (Thanki et al., 2019).

**Figure 4.**
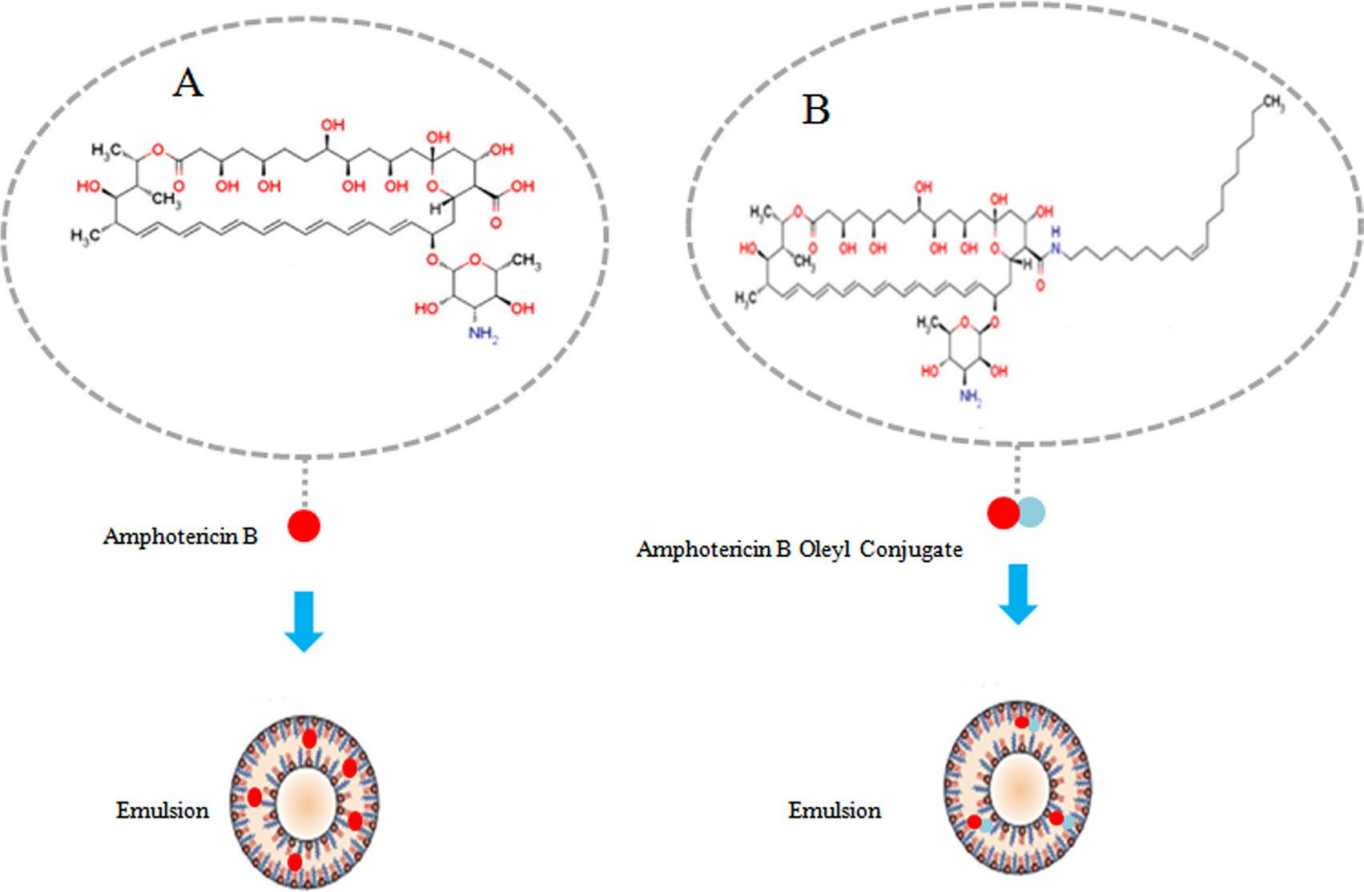
Oral AmB Emulsion. A: AmB was solubilized into NaOH solution or methanol, and then incorporated in emulsion; B: AmB and lipid were congulated, and then incorporated in emulsion.

Interestingly, a novel approach, the benefits of both lipid-drug conjugation and lipid drug delivery, was developed to enhance the oral bioavailability of AmB. In this study, a self-nanoemulsifying drug delivery system (SNEDDS) was designed for oral delivery of AmB-OA (Figure 4) (Thanki et al., 2021). After receiving a 10 mg/kg equivalent AmB dose, AmB-OA SNEDDS formulation resulted in a 8.9-fold higher AUC_Tot_ than that of AmB and 1.8-fold higher AUC_Tot_ than AmB-OA, proving the improvement in the oral bioavailability of AmB through the cumulative effect of AmB-OA loaded SNEDDS (Thanki et al., 2021).

Oral encochleated AmB had a better clinical prospective study, indicating that a novel AmB-loaded cochleates can be attempted via the different preparation methods (Santangelo et al., 2000; Zarif et al., 2000; Delmas et al., 2002; Kibathi et al., 2018; U.S. National Library of Medicine., 2015). Thereby, AmB-lipid conjugation-loaded nanocochleates and emulsions may be a new strategy to develop oral formulation in the future.

## Conclusion

A number of researchers attempt to overcome the oral barriers of poor physicochemical properties of AmB. Unfortunately, the majority of oral AmB formulations are unsuccessful, demonstrating the great difficulty in this task.

Currently, two oral lipid-based formulations of AmB look promising, which has entered clinical development with a favorable outcome of the phase I and II clinical trial. One is oral encochleated AmB developed by Matinas BioPharma (NC, USA), and the other is oral AmB emulsion in development by iCo Therapeutics Inc. (BC, Canada). However, a planned phase II trials in 2020, studying the pharmacokinetics and safety of oral encochleated AmB for prevention of fungal infections in approximately 30 patients undergoing chemotherapy for lymphoblastic leukemia and acute myelogenous, has been withdrawn. iCo Therapeutics in 2020 announce that it will delay the start of the anticipated Phase 2 trial of iCo-019 as the result of uncertainty generated by the current COVID-19 events. As an alternative, AmB-lipid conjugation-loaded lipid drug delivery may be a promising strategy to produce oral formulation in clinical practice.
